# An innovative synthetic support for immunocytochemical assessment of cytologically indeterminate (Bethesda III) thyroid nodules

**DOI:** 10.3389/fendo.2022.1078019

**Published:** 2022-12-01

**Authors:** Silvia Taccogna, Enrico Papini, Roberto Novizio, Martina D’Angelo, Luca Turrini, Agnese Persichetti, Alfredo Pontecorvi, Rinaldo Guglielmi

**Affiliations:** ^1^ Pathology, Ospedale Regina Apostolorum, Albano Laziale, Italy; ^2^ Endocrinology and Metabolism, Regina Apostolorum Hospital, Rome, Italy; ^3^ Endocrinology and Metabolism, Agostino Gemelli University Polyclinic (IRCCS), Rome, Italy; ^4^ Catholic University of the Sacred Heart, Rome, Italy; ^5^ Service of Pharmacovigilance, Regina Elena National Cancer Institute, Hospital Physiotherapy Institutes (IRCCS), Rome, Italy

**Keywords:** thyroid nodule, fine needle aspirate (FNA), immunoistochemestry, indeterminate thyroid cytology, Bethesda III category, Bethesda IV cytology, CytoFoam Core

## Abstract

**Background:**

Fine needle aspiration (FNA) is the procedure of choice in the evaluation of thyroid nodules. Nodules with indeterminate cytological categories, Bethesda III and IV, pose challenges in clinical practice and are frequently submitted to diagnostic surgery. CytoFoam Core (CFCS) uses an absorbent foam device inserted into the needle hub to collect the cytological sample aspirated during FNA. Specimen is formalin-fixed and paraffin-embedded.

**Aim of the study:**

Assessing diagnostic efficacy of CFCS, compared to traditional cytology, in re-evaluating thyroid nodules classified as Bethesda III, using post-surgical histology as reference standard.

**Method:**

Retrospective study on 89 patients with a first indeterminate cytological report who were referred to the Department of Endocrinology of Regina Apostolorum Hospital (Albano L. Rome, Italy) for a second FNA. FNA was performed after at least one month under ultrasound guidance with a 23G needle according to the established procedure. During the second procedure, both traditional cytological (TC) smears and a single-pass CFCS specimen were obtained for each patient. On CFCS samples immunocytochemical staining for Galectin-3, HBME-1, and CK-19 was also performed. 51 patients eventually underwent surgery, and their histological diagnoses were compared to the TC and CFCS reports. Four parameters were evaluated: inadequacy rate, rate of persistent indeterminate (Bethesda III and IV) reports, rate of malignancy in persistently indeterminate nodules, and rate of cancer in lesions cytologically classified as malignant.

**Results:**

Non-diagnostic samples were 6 (11.8%) in TC vs 3 (5.9%) in CFCS (p=0.4). Persistent indeterminate samples were 31 (60.8%) in TC vs 19 (37.2%) in CFCS (p=0.01). Rate of malignancy in persistently indeterminate nodules was 8/19 (42.1%) in CFCS vs 9/31 (29%) in TC group (p=0.3). Nine/51 (17.6%) samples were classified as benign by TC vs 21/51 (41.2%) samples by CFCS (p<0.01). All nodules resulted benign at post-surgical evaluation. Five/51 (9.8%) samples were classified as suspicious for malignancy/malignant in TC group against 8/51 (15.7%) samples in CFCS (p=0.5). Post-surgical evaluation confirmed malignancy in all these cases.

**Conclusion:**

CFCS demonstrated greater diagnostic accuracy than TC in repeat FNA assessment of cytologically indeterminate nodules. CFCS increased the conclusive diagnosis rate and decreased the number of cytologically indeterminate cases.

## Introduction

Thyroid nodules are increasingly detected in clinical practice due to the widespread access to imaging techniques involving the neck. The main issue in their management is to distinguish the minority of malignant lesions, which deserve surgery, from the vast majority of benign thyroid nodules that may be followed over time without intervention (1, 2). Fine needle aspiration (FNA) with ultrasound (US) guidance is the main diagnostic procedure for the assessment of the risk of malignancy of thyroid nodules, being safe, cost-effective and minimally invasive (3, 4). Throughout different studies, from 85% to 90% of US-guided FNA provide a sample adequate for cytological evaluation (5, 6), with a sensitivity ranging from 65% to 98%, a specificity of 72-100% and an accuracy of 84-95% (7). The Bethesda System for Reporting Thyroid Cytopathology (BSRTC) classifies the FNA outcome in 6 diagnostic categories including: (I) non-diagnostic; (II) benign; (III) atypia/follicular lesion of undetermined significance (AUS/FLUS); (IV) follicular neoplasm or suspicious for follicular neoplasm (FN/SFN); (V) suspicious for malignancy; (VI) malignant (8). A non-negligible number of FNA samples are classified as “indeterminate” cytological categories III and IV according to BSRTC, exhibiting a quite wide range of malignancy risk reported in literature, requiring different clinical actions (8, 9). These cytological categories pose a management challenge in clinical practice (9). Even if clinical, laboratory, and US findings offer useful data for refining the risk of thyroid cancer, many of these patients are eventually submitted to diagnostic surgery. According to current thyroid nodule guidelines, either surgery or molecular testing should be considered for patients with Bethesda IV cytology while for Bethesda III nodules a further cytological sampling is recommended. Moreover, managements guidelines are controversial in which surgery, total thyroidectomy and lobectomy, to be performed in AUS/FLUS or FN/SFN, whose management differs among institutions (1, 10, 11). Nevertheless, due to relatively low rate of malignancy revealed by post-surgical histology, the surgical approach may represent an overtreatment in a high number of cases, regardless the type of surgery (12).

Molecular testing can be employed to improve the accuracy of preoperatory diagnosis in thyroid nodules with indeterminate cytological report. Currently, multi-gene classifiers offer relevant sensitivity and high negative predictive value (NPV) but are still limited by a relatively low specificity and positive predictive value (PPV) (13). Most important, the elevated cost of these techniques make their routine use in clinical practice as extremely expensive for the National Health Services (NHS). Thus, at variance with their diffusion in the USA, only few centers in Europe regularly perform molecular testing as a routine complement to the diagnostic work up of class III and IV cytological samples (14, 15).

Immunohistochemical studies may provide complementary information about the nature of thyroid FNA samples (7). These tests are rather inexpensive and can be routinely performed in pathology departments. Main limitations of traditional procedures are the modalities of processing of the samples, which require working time and specific skill from the operators, the uneven distribution and the possible loss of thyroid cells, and the potentially inadequate staining of intracellular antigens (16).

The CytoFoam Core system (CFCS) is proposed as an innovative technique that can provide optimal formalin-fixed and paraffin-embedded (FFPE) cytologic specimens, obtained with a single FNA pass according to the established sampling procedure (3). The CFCS samples are suitable for high quality immunohistochemical studies, which are performed without destruction of the cytological material that remains available for further studies.

Aim of the present study was to assess the technical feasibility and the cost of CFCS and its diagnostic accuracy in cytologically indeterminate thyroid nodules. Outcomes were compared to the results of traditional cytology, with the use of post-surgical histology as the reference standard.

## Methods

### Design of the study

Retrospective single center blinded study. Ethical review and approval were not required for the study on human participants in accordance with the local legislation and institutional requirements. Written informed consent for participation was not required for this study in accordance with the national legislation and the institutional requirements.

### Patients

**Figure 1:**
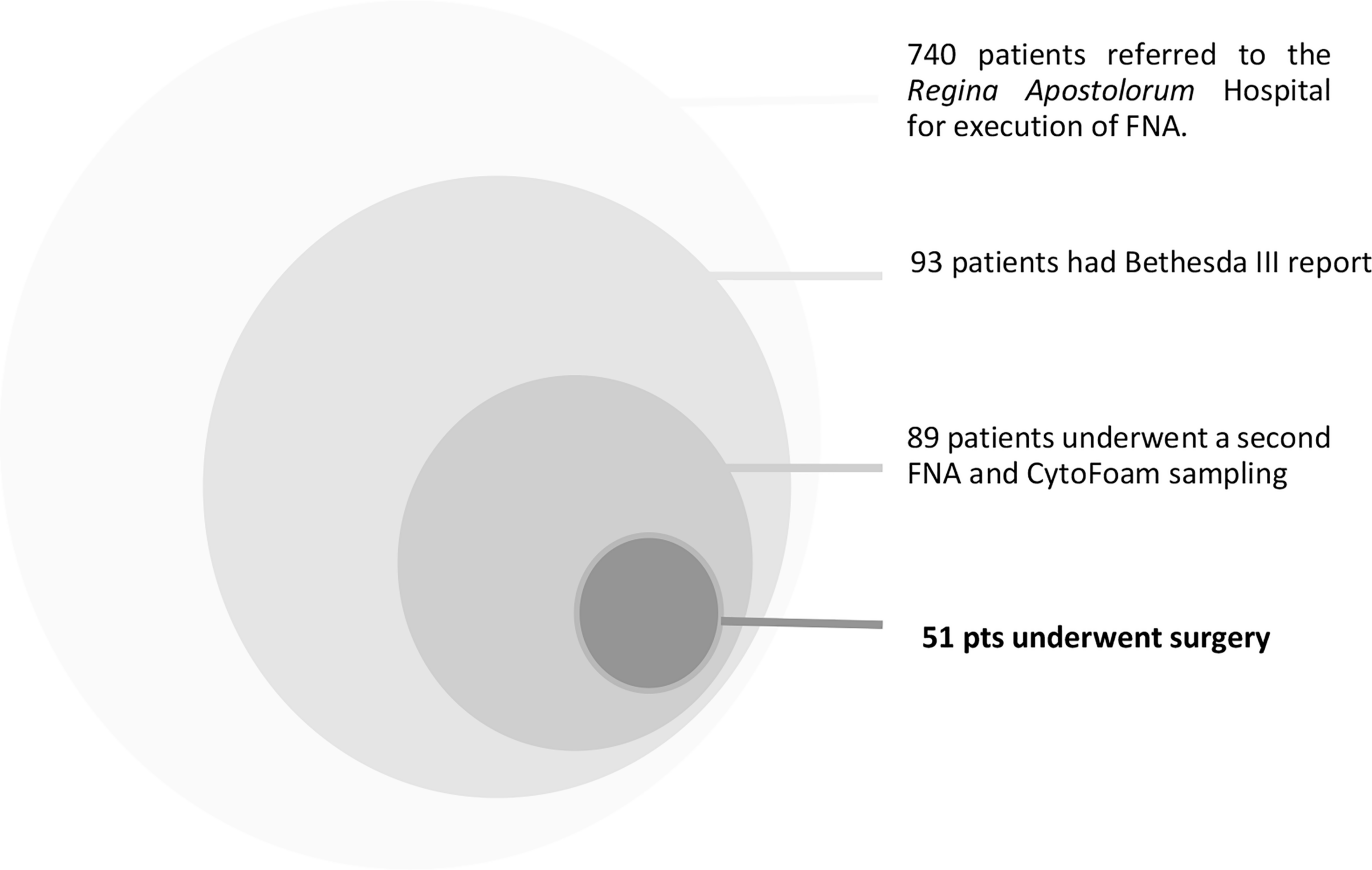
Patients Selection.

From June 2019 to June 2020, 740 patients with solid not hyperfunctioning thyroid nodules were referred for FNA assessment to the thyroid clinic of the Department of Endocrinology of Regina Apostolorum Hospital, Albano, Rome. All sampling procedures were performed by two endocrinologists (EP, RG) and were examined in the Pathology Department of the same hospital by two experienced pathologists (ST and LT). FNA was performed with 23- or 25-gauge needles and the aspirated material was spread on 6 slides which were stained with both Papanicolaou and May-Grunwald Giemsa methods (16). Ninety-three patients (12.5%) had a low-risk indeterminate cytological report (Bethesda class III or Tir3A category, according to the Italian SIAPEC-AME-AIT-SIE classification) (5). According to current thyroid clinical practice guidelines, a second FNA was performed after 1 - 3 months for the definition of patients’ clinical management (1). During the second FNA procedure, an additional sampling was performed using the CFCS in 89 patients. Fifty-one patients who eventually underwent surgery because of suspicious cytology at second evaluation, compressive or cosmetic symptoms, anxiety for malignancy risk, or suspicious clinical or US data were included in our retrospective study (**Figure 1**).

### Cytofoam core procedure

**Figure 2:**
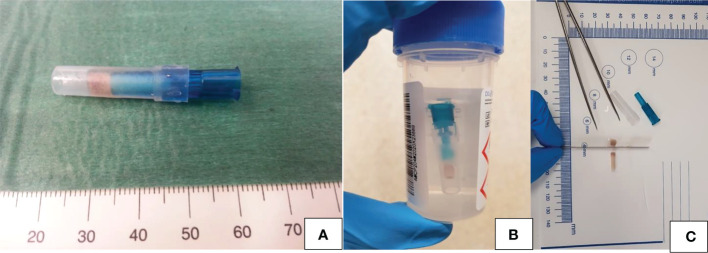
**(A–C)** Cytofoam core sampling: schematic illustration of the sample and of its processation. **(A)** Foam structure of the CFCS protected by a plastic guard cap before fixation; **(B)** CFCS sample fixation in 10% neutral buffered formalin; **(C)** Sections of the CFCS sample ready to be embedded in a paraffin block.

Samples for the CFCS procedure were collected with a dedicated US-guided FNA, performed according to the standard procedure (17). An 8 x 3 mm cylinder of synthetic foam with elevated absorbent structure (Diapath, Martinengo company, Italy) was inserted between the hub of a 23 G/25 G needle and the aspirating syringe (**Figure 2**). The foam structure worked as a terrycloth, holding the cellular material aspirated during the FNA biopsy. After a 10 – 15 seconds aspiration, the foam core was removed by the needle hub, protected with a plastic guard cap, and placed into 10% neutral buffered formalin for 12 hours. Once fixed, the foam core was pulled from the adaptor, automatically processed, and embedded in paraffin blocks. Then, four sections were obtained and prepared to be studied: the first section was stained with hematoxylin-eosin for morphologic evaluation, the others automatically treated for immunohistochemistry in a Dako Autostainer (Dako, Carpinteria, CA, USA) with antibodies for Galectin 3 (dilution 1:50, clone 9C4, Cell Marque), Cytokeratin 19 (ready to use, clone RCK 108 Dako Corporation, Carpentaria, California) and HBME1 (dilution 1:50, clone M3505, Dako Corporation, Carpentaria, California). The staining was completed using a streptavidin-biotin-complex detection method (LSAB2). The remaining material was stored for further possible examinations.

Surgical specimens were fixed in 10% buffered formaldehyde and embedded in paraffin, and 5-mm-thick microtome sections were stained with haematoxylin-eosin. Cytologic specimens and histologic sections were separately and blindly reviewed by two experienced pathologists of our center.

## Study methodology

Thyroid CFCS specimens were analyzed by the two pathologists, and, for each patient, the results were blindly compared to those of the traditional cytological smears.

**Figure 3:**
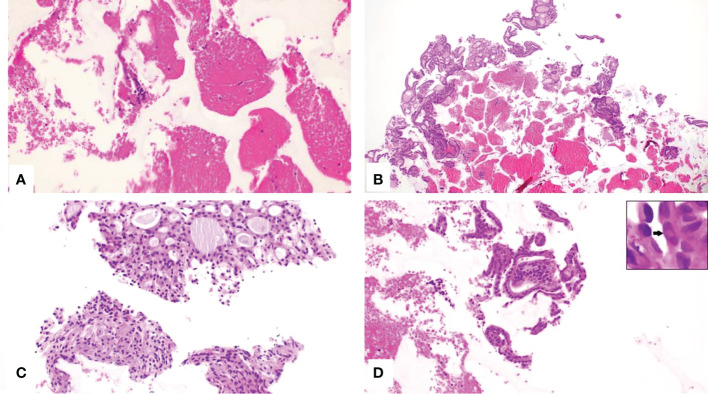
**(A–D)**, CytoFoam Core Quality Classification. Hematoxilin & Eosin staining of the CFCS Histology, (H&E)x200: **(A)** Non-diagnostic sample due to the insufficient number of cells (Class I); **(B)** Sample from a benign thyroid nodule showing an adequate number of colloid containing follicles (Class II); **(C)** Indeterminate sample showing an adequate number of thyrocytes, mainly organized in microfollicular structures and with minor cellular atypia (Class III); **(D)** Tissue fragment with groups of irregular cells. The nuclei are variably enlarged with intranuclear inclusions (arrow inset) (Class IV).

A four-class categorization of CFCS cytological findings was arbitrarily built. Class I identified non-diagnostic samples, class II included samples with benign characteristics, class III identified indeterminate samples, and class IV included samples with characteristics suspicious for malignancy (**Figure 3**). Specifically, CFCS samples were defined as: non diagnostic when the cell number or quality was insufficient for a reliable diagnosis; benign when architectural or cellular atypia were absent and the three immunocytochemical markers were consistently negative; as indeterminate when minimal architectural or cellular atypia were present and the immunocytochemical markers were partially (1-2 out of 3) positive; malignant when architectural or cellular atypia were severe and/or all three immunocytochemical markers were positive.

Post-surgical histology was used as the reference standard for the final diagnosis of the nodules under investigation.

The following parameters of diagnostic efficacy were analyzed: (I) percentage of CFCS non-diagnostic samples; (II) percentage of CFCS samples reported as benign which were confirmed as benign at histology; (III) percentage of persistently indeterminate samples; (IV) percentage of samples reported as indeterminate which resulted as malignant at histology; (V) percentage of samples reported as malignant which were confirmed as malignant at post-surgical histology. The diagnostic efficacy of traditional cytology and CFCS system were compared on the base of the final histologic report.

## Statistical analysis

Data were collected on a Microsoft Excel database. X-square and, when appropriate, exact Fisher’s test were used to compare results in traditional cytology group vs CFCS group. The level of significance was set at α < 0.05. Data analysis was performed using SPSS v22 (IBM). An external monitor independently processed the data.

## Results

### Traditional cytology reports & results of CytoFoam categories vs. post-surgical histology

Four out of 6 (66.6%) non-diagnostic specimens with the second TC resulted as malignant at histology versus 2 out of 3 (66.6%) of those with CFCS. 9 sample were classified as benign through traditional cytology, while 21 through CFCS: all classified as benign at post-surgical histology. Other diagnostic outcomes of CFCS and of traditional cytology are compared to the results of postsurgical histology in **Tables 1**, **2**.

**Table 1:** Traditional cytology reports vs. post-surgical histology.

Cytological diagnosis	Number of reports	Benign at post-surgical histology N (%)	Malignant at post-surgical histology N (%)
Bethesda I	6	2/6 (33.3%)	4/6 (66.6%)
Bethesda II	9	9/9 (100%)	0/9 (0%)
Bethesda III	22	15/22 (68.2%)	7/22 (31.8%)
Bethesda IV	9	7/9 (77.8%)	2/9 (22.2%)
Bethesda V	4	0/4 (0%)	4/4 (100%)
Bethesda VI	1	0/1 (0%)	1/1 (100%)

**Table 2:** CytoFoam Core System reports vs. post-surgical histology.

CytoFoam Class	Number of reports	Benign at post-surgical histologyN (%)	Malignant at post-surgical histologyN (%)
I	3	1/3 (33.3%)	2/3 (66.6%)
II	21	21/21 (100%)	0/21 (0%)
III	19	11/19 (57.9%)	8/19 (42.1%)
IV	8	0/8 (0%)	8/8 (100%)

### Comparison of the results of CytoFoam categories vs the traditional cytology classes

The occurrence of non-diagnostic samples (Bethesda I) was 6/51 (11.8%) with TC vs 3/51 (5.9%) with CFCS (p=0.4).

Nine of 51 (17.6%) samples were classified as benign (Bethesda II) with TC vs 21/51 (41.2%) with CFCS (p<0.01).

Persistent indeterminate samples (Bethesda III and IV) were 31/51 (60.8%) with TC vs 19/51 (37.2%) with CFCS (p=0.01).

Five of 51 samples (9.8%) were classified as suspicious for malignancy or malignant (Bethesda V and VI) with TC versus 8/51 (15.7%) samples in the CFCS group (p=0.55). (**Table 3**)

**Table 3:** Comparison of the results of CytoFoam categories vs the traditional cytology classes.

CytoFoam Category	BSRTC	TC	CFCS	P-value	Post -surgical histology
I	Bethesda I	6 (11.8%)	3 (5.9%)	0.4 *	/
II	Bethesda II	9 (17.6%)	21 (41.2%)	<0.01	33
III	Bethesda III - IV	31 (60.8%)	19 (37.2%)	0.01	/
IV	Bethesda V-VI	5 (9.8%)	8 (15.7%)	0.5 *	18

TC: traditional cytology; CFCS: cytofoam.

*Exact Fisher’s test.

### Traditional cytology vs. cytofoam. Rate of inadequate and indeterminate reports and rate of malignant histology in indeterminate, suspicious or malignant reports

All specimens classified as benign with traditional cytology or with CFCS resulted as benign at post-surgical histological evaluation (benign rate, 100%).

The rate of malignancy in persistently indeterminate nodules was 8/19 (42.1%) in CFCS group vs 9/31 (29%) in TC group (p=0.3).

All specimens classified as malignant with TC (Bethesda V or VI) or with CFCS (IV) resulted as malignant at post-surgical evaluation (malignancy rate, 100%). (**Table 4**)

**Table 4:** Traditional cytology vs cytofoam. Rate of inadequate and indeterminate reports and rate of malignant histology in indeterminate, suspicious or malignant reports.

Report	Traditional Cytology	CytoFoam	P-value
Rate of malignant histology in cytological benign reports	0/9 (0%)	0/21 (0%)	/
Rate of malignant histology in cytological indeterminate reports	9/31 (29%)	8/19 (42.1%)	0.3 *
Rate of malignant histology in cytological suspicious or malignant reports	5/5 (100%)	8/8 (100%)	/

*Exact Fisher’s test.

### Traditional cytology vs. cytofoam. Rate of conclusive reports

As a whole, out of the 51 nodules classified as Bethesda III at initial cytological assessment, 14 (27.4%%) had a conclusive diagnosis, either benign or malignant, with the second cytological sample versus 29 (56.8%) with the CFCS specimen (p=0.002). (**Table 5**)

**Table 5:** Traditional cytology vs cytofoam. Rate of conclusive reports.

Report	Traditional Cytology	CytoFoam	P-value
Rate of conclusive reports	14/51 (27.4%)	29/51 (56.8%)	<0.01

The use of CFCS has statistically significantly increased the number of cytologically benign reports, Moreover, the number of indeterminate was statically reduced, with an increase in the rate of conclusive report, while conserving 0% of rate of malignant histology in cytological benign reports and 100% of rate of malignant histology in cytological suspicious or malignant reports, so not influencing the negative predictive value and positive predictive value among cytologially benign or malignant sample, respectively. Results of all the Bethesda III patients’ re-assessment are shown in **Table 6**. Relevant results are summarized in **Table 7**.

**Table 6:** Results of the Bethesda III patients’ re-assessment. Cytological diagnosis vs. immunocytochemical classes.

ID PTS	Bethesda Category	HMBE 1	Galectin 3	Cytokeratin 19	CytoFoam Class	M vs B
1	III	0	0	0	3	1
2	V	2	2	2	4	1
3	II	1	1	1	2	0
4	I	1	1	1	2	0
5	I	1	1	2	3	1
6	IV	1	1	1	2	0
7	IV	1	1	2	3	0
8	V	2	2	2	4	1
9	IV	1	1	2	3	1
10	IV	1	1	1	2	0
11	II	1	1	1	2	0
12	IV	1	1	2	2	0
13	II	0	0	0	2	0
14	II	1	1	1	2	0
15	IV	1	1	1	2	0
16	IV	1	2	2	3	1
17	IV	2	1	2	3	1
18	IV	2	1	1	3	0
19	V	2	2	2	4	1
20	IV	2	1	2	3	0
21	IV	1	1	1	2	0
22	IV	0	0	0	1	0
23	IV	2	1	2	3	0
24	IV	2	2	2	4	1
25	II	2	1	2	3	0
26	III	2	1	2	3	0
27	III	1	1	2	2	0
28	III	1	2	2	3	0
29	V	2	2	2	4	1
30	III	1	1	2	2	0
31	VI	2	2	2	4	1
32	III	1	1	2	3	1
33	III	1	1	2	3	1
34	III	1	1	1	2	0
35	III	2	1	1	2	0
36	I	2	1	1	3	1
37	II	1	1	1	2	0
38	I	2	2	2	4	1
39	I	1	1	2	2	0
40	III	2	1	2	2	0
41	III	1	1	1	2	0
42	IV	2	1	2	3	0
43	III	1	1	2	1	1
44	I	1	2	1	4	1
45	III	0	0	0	1	1
46	II	1	1	1	2	0
47	II	1	1	1	2	0
48	II	2	1	2	3	0
49	II	2	1	2	3	0
50	III	1	1	2	3	0
51	III	1	1	1	2	0

From left to right: id pts, identification code of the patient; SIAPEC-AME-AIT-SIE report; HMBE1 evaluation, where 0 non-evaluated or non-diagnostic, 1 means negative, 2 means positive; Galectin 3 evaluation, where 0 non-evaluated or non-diagnostic, 1 means negative, 2 means positive; Cytokeratin 19 evaluation, where 0 non-evaluated or non-diagnostic, 1 means negative, 2 means positive; CytoFoam class report; M (malignant) vs B (benign), where 0 means a benign histology report, while 1 means a malignant histology report.

**Table 7:** Statistically relevant results of CytoFoam categories vs traditional cytology classes.

CytoFoam Category	Cytology	TC	CFCS	P-value
II	Bethesda II	9 (17.6%)	21 (41.2%)	<0.01
III	Bethesda III - IV	31 (60.8%)	19 (37.2%)	0.01
**Report**				
Rate of malignant histology in cytological benign reports		0/9 (0%)	0/21 (0%)	/
Rate of malignant histology in cytological suspicious or malignant reports		5/5 (100%)	8/8 (100%)	/
Rate of conclusive reports		14/51 (27.4%)	29/51 (56.8%)	<0.01

## Immunocytochemical staining

The quality of the immunocytochemically stained samples was arbitrarily classified as follows: 0 when the staining was negative, 1 when positive, and 2 when inadequate for evaluation. Based on this classification, the immunocytochemically stained samples were rated as negative in 14 cases (27.4%), as positive in 33 cases (64.7%), and as inadequate in 4 cases (7.8%).

### Complications

No local anaesthesia was requested. No major nor minor complications were reported with the two procedures and no patient required post-procedural clinical or US observation. Pain was described as well tolerated and, according to a 1 to 10 visual analogue scale, was classified as a mean of 2 with the conventional FNA (range 2 to 4) and as 3 (2 to 5) with the CFCS procedure (p = 0.8). No postprocedural medication was necessary with both the procedures.

### Time

The time employed for the two sampling procedures was similar, with a range from 15 to 30 seconds.

## Discussion

Despite the advances in US imaging, especially through artificial intelligence systems and lately the possible application of contrast in ultrasound, and the promising results of molecular analysis, the main diagnostic step for thyroid nodules still relies on FNA results (5, 18, 19). FNA procedure is minimally invasive, performed with negligible patient discomfort, and offers an elevated diagnostic accuracy (3). However, a considerable percentage of thyroid cytological samples do not reach a conclusive diagnosis and are classified as non-diagnostic or at indeterminate risk of malignancy (20). According to current thyroid nodule guidelines, either surgery or molecular testing should be considered for patients with Bethesda IV cytology while for Bethesda III nodules a further cytological sampling is recommended (1). In case of a persistent Bethesda III cytological diagnosis either the assessment of molecular markers, active surveillance or surgery are suggested, based on the clinical condition, the local resources, and the patient preferences. Presently, a non-negligible percentage of patients with persistent Bethesda III diagnosis eventually undergo thyroid surgery, generally for diagnostic purpose. The risk of malignancy for Bethesda III thyroid nodules reported in literature shows a a quite wide range, from 19% to 55% in populations with environmental risk factors (e.g. endemic goiter) (4, 21–24). In the majority of these cases, surgery results in a benign lesion at histology but thyroidectomy carries a non-negligible risk of complications, increases healthcare cost and may negatively influence the quality of life of patients (4).. Most patients could then benefit more from a watchful waiting rather than from surgery. Thus, the use of ancillary tests is advocated to allow a more accurate pre-operatory stratification of the risk of malignancy and decrease the still elevated number of diagnostic thyroid surgeries.

Immunohistochemistry (ICC) was introduced in the 1970s as a diagnostic tool for both surgical pathology and cytopathology (16). While ICC plays a relevant role in the differential diagnosis between follicular and C cell-derived neoplasms and in the identification of primary or metastatic thyroid neoplasms, its usefulness in the pre-operatory assessment of risk of malignancy in follicular-patterned lesions is still unsettled (1, 25). Results from histological specimens may be discrepant from those obtained from cytological samples due to differences in fixatives, fixation methods, and/or antigen activation treatment. Immunostaining of histological specimen is carried out using formalin-fixed, paraffin-embedded, tissues with or without antigen retrieval. On the other hand, immunostaining of FNA smears is usually carried out using 95% alcohol-fixation, followed by a further fixation with phosphate-buffered formalin solution, and by an antigen activating treatment (16). Due to the low reliability of alcohol-fixation for the staining of target antigens, an additional formalin fixation (double fixation) is generally needed and it is followed by the antigen activating treatment. Formalin fixation and antigen activation treatment provide reliable results, but the process may result in loss of cell material and deterioration of the sample under examination. These supplementary investigations are best performed on cell blocks, but cell-block preparation is burdensome in high-volume laboratories. All these factors have until now limited the use of immunocytochemistry as a complement to the morphological diagnosis in indeterminate cytological samples.

The combination of CK-19, HBME-1 and galectina-3 immunocytochemistry is the most useful ancillary technique for improving the differential diagnosis in follicular-derived thyroid nodules. Notably, these markers show a diffuse reactivity in true malignant lesions (follicular cancer, classical variant of papillary cancer, and follicular variant of papillary cancer) while a focal staining is observed in benign neoplastic (follicular adenoma) and benign non-neoplastic (nodular goiter) lesions. In various studies, the sensitivity of CK19, galectin-3, HBME-1 was 75.41%, 88.52% and 71.31% respectively, and the specificity of CK19, galectin-3 and HBME-1 was 70.89%, 64.56% and 84.81% (26). The aim of the CFCS testing is to improve the diagnostic accuracy of FNA for guiding clinical action in cytologically indeterminate nodules. Only three diagnostic classes were considered for operative purposes: benign, indeterminate, and probably malignant. Consequently, persistently indeterminate nodules (Bethesda III-IV) are included in the CFCS class III and suspicious/neoplastic nodules (Bethesda V and VI) in the CFCS class IV. This simplified classification can be achieved because the evaluation of thyroid nodules’ risk of malignancy is based on both morphological criteria and immunohistochemistry results. The results from our study demonstrate that the use of CFCS provides high quality immunocytochemical staining in the vast majority (92%) of cytological samples. When compared to traditional cytology, CFCS provides an increase, from 27.4 to 56.8%, in the number of conclusive diagnosis obtained with repeat FNA sampling. The predictive value of the immunophenotypic assessment is confirmed by the elevated concordance of the cytological diagnosis with the final post-surgical assessment (100% concordance for both benign and malignant diagnosis). Notably, when all the three markers were negative, the NPV for thyroid cancer was 100% providing a reliable rule-out test. Three suspected cases classified at CFCS class III (cases #25-48-49), resulted at histological examination to be neoplasms, even if non-malignant ones (one follicular adenoma and three NIFTP). So, the surgical indication provided by CFCS examination may be considered as appropriate.

These favorable outcomes are mostly due to the sample characteristics, that are similar to those of a micro-histological specimen and are comparable to those obtained by the more expensive, invasive, and difficult-to-perform core-needle biopsy. Importantly, the immunophenotypic evaluation does not induce any deterioration of the specimen during the staining procedure. On clinical grounds, the CFCS procedure does not require additional time or any observation period when compared to the traditional FNA biopsy and is well tolerated by the patients. The increase in costs of this malignancy rule-out test is modest, as the price of the CFCS device is about 8 euros (27).

In conclusion, this feasibility study demonstrates that cyto-foam core technique is a simple, safe, inexpensive, and reproducible procedure. The short processing time and the use of routine technical resources make this modality of immunocytochemical assessment of thyroid FNA samples suitable for routine use in most pathology laboratories. When testing of molecular markers is not accessible, immunocytochemical staining with the use of CFCS may provide - in addition to the clinical, laboratory, and US data - a further relevant element in the multifactorial choice of either surgical resection or follow-up for thyroid nodules with indeterminate cytology. Low numerical sample appears to be the main limitation of the study.

## Data availability statement

The original contributions presented in the study are included in the article/supplementary material. Further inquiries can be directed to the corresponding author.

## Ethics statement

Ethical review and approval was not required for the study on human participants in accordance with the local legislation and institutional requirements. Written informed consent for participation was not required for this study in accordance with the national legislation and the institutional requirements.

## Author contributions

All authors listed have made a substantial, direct, and intellectual contribution to the work and approved it for publication.

## Funding

The authors declare that this study received funding from Diapath S.p.A. The funder was not involved in the study design, collection, analysis, interpretation of data, the writing of this article, or the decision to submit it for publication.

## Conflict of interest

The authors declare that the research was conducted in the absence of any commercial or financial relationships that could be construed as a potential conflict of interest.

## Publisher’s note

All claims expressed in this article are solely those of the authors and do not necessarily represent those of their affiliated organizations, or those of the publisher, the editors and the reviewers. Any product that may be evaluated in this article, or claim that may be made by its manufacturer, is not guaranteed or endorsed by the publisher.
